# Advancing epileptic seizure recognition through bidirectional LSTM networks

**DOI:** 10.3389/fncom.2025.1668358

**Published:** 2025-10-17

**Authors:** Sanaa Al-Marzouki

**Affiliations:** Department of Statistics, Faculty of Science, King Abdul Aziz University, Jeddah, Saudi Arabia

**Keywords:** epileptic seizure recognition, bidirectional LSTM, deep learning, EEG analysis, neural networks, healthcare technology, neurological disorders, brain stimulation

## Abstract

Seizure detection in a timely and accurate manner remains a primary challenge in clinical neurology, affecting diagnosis planning and patient management. Most of the traditional methods rely on feature extraction and traditional machine learning techniques, which are not efficient in capturing the dynamic characteristics of neural signals. It is the aim of this study to address such limitations by designing a deep learning model from bidirectional Long Short-Term Memory (BiLSTM) networks in a bid to enhance epileptic seizure identification reliability and accuracy. The dataset used, drawn from Kaggle’s Epileptic Seizure Recognition challenge, consists of 11,500 samples with 179 features per sample corresponding to different electroencephalogram (EEG) readings. Data preprocessing was utilized to normalize and structure the input to the deep learning model. The proposed BiLSTM model employs sophisticated architecture to leverage temporal dependency and bidirectional data flows. It incorporates multiple dense and dropout layers alongside batch normalization to enhance the capability of the model in learning from the EEG data in an efficient manner. It supports end-to-end feature learning from the raw EEG signals without the need for intensive preprocessing and feature engineering. BiLSTM model performed better than others with 98.70% accuracy on the validation set and surpassed traditional techniques. The F1-score and other statistical metrics also validated the performance of the model as the confusion matrix achieved high values for recall and precision. The results confirm the capability of bidirectional LSTM networks to better identify seizures with significant improvements over conventional practices. Apart from facilitating seizure detection in a reliable fashion, the method improves the overall field of biomedical signal processing and can also be used in real-time observation and intervention protocols.

## 1 Introduction

Epilepsy is a chronic neurological disorder characterized by the recurrence of unprovoked seizures in approximately 50 million individuals worldwide. Early and accurate seizure detection is a significant parameter to be considered in appropriate management and treatment, enhancing the quality of life and safety of the patients. Visual detection of seizures has been done using electroencephalograms (EEGs), but it is not only time-consuming but also prone to human errors due to the EEG signal being complex and subtle in nature.

The limitations of the traditional EEG analysis such as relentless human intervention and knowledge to correctly interpret have compelled the development towards autonomous systems. Traditional machine learning (ML) models have been utilized in this case to make it possible, with over-feature extraction and engineering to pipe through the EEG data. They are unable to tap temporal dynamics and EEG high-dimensional structure, leading to performance deterioration in real-world scenarios.

Deep learning techniques, i.e., long short-term memory (LSTM) networks, have been found to be useful tools in such case (Chhajer et al., 2022). LSTMs can learn temporal patterns over long-time frames and hence are very well-suited for modeling EEG signals since EEG signals are temporal and non-linear.

Existing seizure detection models continue to be afflicted by deep issues such as low accuracy, failure to generalize across patients, and heavy reliance on hand-designed features that introduce model complexity and training bias. These issues underscore the need for models that can learn to discover patterns from raw data with little preprocessing.

Bidirectional LSTM (BiLSTM) networks extend traditional LSTMs with the added advantage of providing additional context from the sequence data, with processing in forward and backward directions. The two-path architecture adds more capability for the model to learn from temporal data, which can result in better and stronger seizure detection. Although they are promising, the application of BiLSTMs in the classification of EEG signals is yet to be explored, and thus the research gap is important and this research will seek to bridge that gap.

This work contributes to the detection of epileptic seizures using the following innovations:

1. Creating a novel deep learning approach using bidirectional LSTM networks to enhance the performance of detecting epileptic seizures from EEG signals.
2. Using a publicly available, annotated dataset to train and test the new model to enable reproducibility and reliability in real-world applications.
3. Testing the model using various metrics like accuracy, confusion matrix, F1-score, and other statistical parameters applicable to the task to demonstrate its performance and improvements from the existing state.
4. Comparing the BiLSTM model to traditional machine learning and other deep learning methods to determine its strengths and possible areas of research.

Through these objectives, the study aims to not only improve the technology of seizure detection but also to contribute to the overall discussion of the application of deep neural network architectures in health informatics.

While hybrid CNN-BiLSTM architectures offer the promise of capturing both spatial and temporal EEG features, this study prioritizes establishing a robust temporal modeling baseline through BiLSTM networks. Future investigations will evaluate CNN-BiLSTM combinations to harness complementary spatial information from multi-channel EEG recordings.

This study differentiates itself from existing BiLSTM-based seizure detection research through its rigorous data preprocessing strategy that retains raw EEG temporal characteristics without synthetic augmentation, a carefully tuned dropout-batch normalization combination that enhances generalization, and systematic use of Sparse Categorical Cross entropy to align with integer-based labels. Also, an ablation study has been conducted to evaluate hyperparameter sensitivity and demonstrate the stability of the proposed architecture, establishing a robust and reproducible benchmark for future studies.

The manuscript is structured to provide clear and logical progression of the research. Section 2, “Literature review,” critically assesses existing studies, setting the context for the advancements introduced by the approach. In Section 3, “Methodology,” detail of experimental setup, including data preprocessing and the specifics of the BiLSTM model architecture, ensuring reproducibility and clarity in the approach is presented. Section 4, “Results and discussion,” presents the findings, where the efficacy of the model is analyzed and compared against traditional methods, using robust statistical metrics to underscore the improvements. This structured approach not only enhances the understanding of an innovative model but also solidifies its potential application in clinical settings.

## 2 Related work

Standard machine learning techniques such as Support Vector Machines (SVMs), Decision Trees, and k-Nearest Neighbors (k-NN) have been seminal in seizure detection application (Pan et al., 2025). SVMs have particularly been noted as being strong in high-dimensional feature spaces, prevalent in EEG signals. However, the methods at times are vulnerable to optimal feature extraction and selection, which may be time-consuming and does not capture all dynamic features of EEG signals. Decision Trees and k-NN have provided interpretable models but are overfitted and do not generalize to other objects (Pan et al., 2024).

Deep learning has introduced a significant shift in seizure detection techniques. Convolutional Neural Networks (CNNs) have been applied to segments and automatically extract spatial hierarchies of features from raw EEG signals (Chaudhary et al., 2024). Recurrent Neural Networks (RNNs) and their variants like LSTMs have addressed the temporal aspects of EEG data by effectively learning dependencies in time-series information (Ma et al., 2024). These models have shown improved accuracy over classical methods by autonomously learning features directly from the data, reducing the need for manual feature engineering. Table 1 illustrates the study of existing techniques in the related field.

**Table 1:** Literature review.

Study	Objectives	Remarks
Wong et al. (2023)	Review of EEG datasets for seizure detection and prediction using ML techniques	Discusses the variability in dataset formats and structures, highlighting the need for standardized guidelines to enhance reproducibility and generalizability in seizure detection algorithms
Handa et al. (2023)	Review and comparison of EEG datasets for epilepsy diagnosis and seizure detection	Emphasizes the evolution of dataset availability and suggests the need for a standardized protocol to improve AI applications in epilepsy diagnosis
Tran et al. (2022)	Introduce a machine learning approach for epileptic seizure detection from EEG signals	Proposes a novel method involving feature selection and reduction to enhance the efficiency of seizure detection models
Hassan et al. (2022)	Develop an automatic seizure detection system combining CNN and traditional machine learning classifiers	Utilizes feature extraction and selection to address the challenges of complex EEG signal analysis for seizure detection
Ahmad et al. (2024)	Present an efficient feature selection and classification method for EEG-based epileptic seizure detection	Introduces an explainable AI approach to improve seizure detection and the interpretability of AI decisions in clinical settings
Abdulwahhab et al. (2024)	Explore deep learning techniques for seizure detection using EEG signal analysis	Combines CNN and RNN models to analyze time-frequency transformed EEG data for high accuracy in seizure detection
Chen et al. (2023)	Propose a CNN-based automated detection system for EEG signals using feature fusion	Highlights the advantages of combining multiple feature types to enhance classification accuracy in epilepsy detection
Liu et al. (2023)	Investigate the significance of periodic and aperiodic EEG components for seizure detection and prediction	Demonstrates the importance of differentiating EEG components to improve the accuracy of epilepsy detection methods
Varlı and Yılmaz (2023)	Develop a combined deep learning model for epilepsy diagnosis and seizure detection	Uses a mix of image and numerical data from EEG signals to classify and detect epileptic activity across multiple datasets
Hassan et al. (2022)	Improve epileptic seizure detection using a novel feature selection method and neural network classifier	Employs empirical mode decomposition and a feature selection algorithm to refine the inputs for neural network-based seizure detection

Bidirectional LSTMs (BiLSTMs) extend the capabilities of LSTMs by processing data in both forward and reverse directions, which helps in capturing context more effectively. While there have been applications of BiLSTMs in other domains such as natural language processing and speech recognition, their use in EEG signal classification is less explored (Albalawi et al., 2024). Existing studies utilizing BiLSTMs for EEG have demonstrated potential in enhancing model performance by leveraging both past and future context of the EEG sequence, yet often these applications do not fully exploit the bidirectional architecture’s capability to enhance the temporal resolution.

Existing research on EEG-based seizure detection has predominantly focused on either feature-engineered classical methods or unidirectional deep learning models (Yin et al., 2025). This work extends the current literature by implementing a BiLSTM approach, which inherently learns to recognize and classify seizure and non-seizure events from raw EEG data without the necessity for pre-defined feature extraction. The proposed model not only addresses the limitations of temporal context capture in traditional LSTM models but also showcases a significant improvement in detection performance, demonstrating the BiLSTM’s ability to understand the complex, nonlinear interdependencies of EEG signals more effectively than previously reported methods. By intensive testing, analyzing, and verifying, this research confirms the stability and consistency of BiLSTMs and presents an efficient and scalable approach to analyzing real-time seizure detection systems.

Recent advances in seizure detection using EEG are centered on the use of attention mechanisms to improve feature representation and increase explainability. Attention layers, together with recurrent architectures, have been proven to selectively focus on relevant temporal or spatial segments of EEG, improving classification performance and providing clinically meaningful results. Architectures such as Attention-based BiLSTMs and Transformer-based architectures already demonstrate promising performance on publicly available EEG datasets and suggest that the incorporation of attention will offer additional performance and interpretability boost for seizure detection systems.

## 3 Methodology

**Figure 1:**
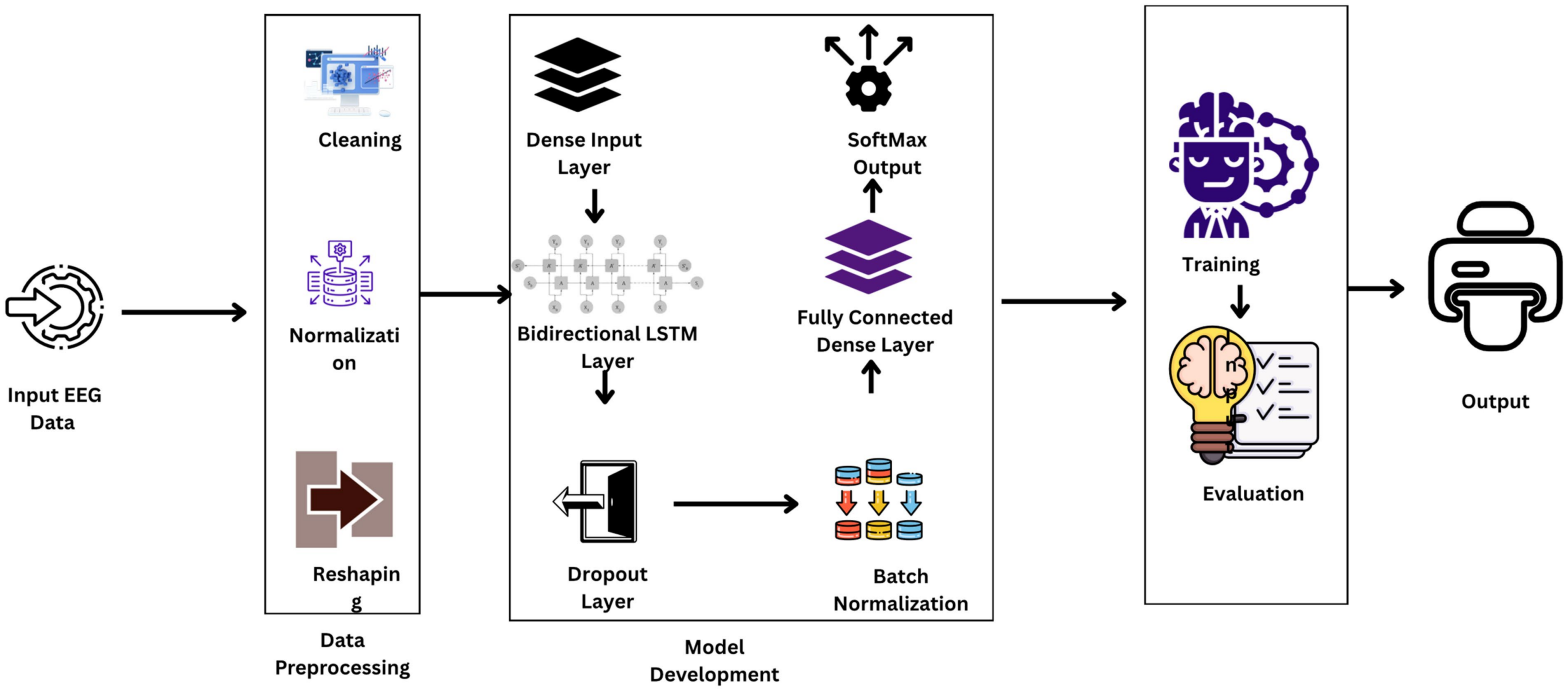
Workflow diagram of the proposed model.

In the present study, the Kaggle Epileptic Seizure Detection dataset with EEG signals of 500 patients was used to train an aggressive BiLSTM model for seizure detection. The data were normalized and scaled and split into the training set, validation set, and test set. BiLSTM consists of layers like Dense, Bidirectional LSTM, Dropout, and Batch Normalization to efficiently process temporal EEG signals. Training was up to 100 epochs with methods such as early stopping and model checkpointing to improve performance and avoid overfitting. This holistic method is applied with the goal of improving seizure detection system prediction significantly and the process of this holistic method is shown in Figure 1.

### 3.1 Dataset description

**Figure 2:**
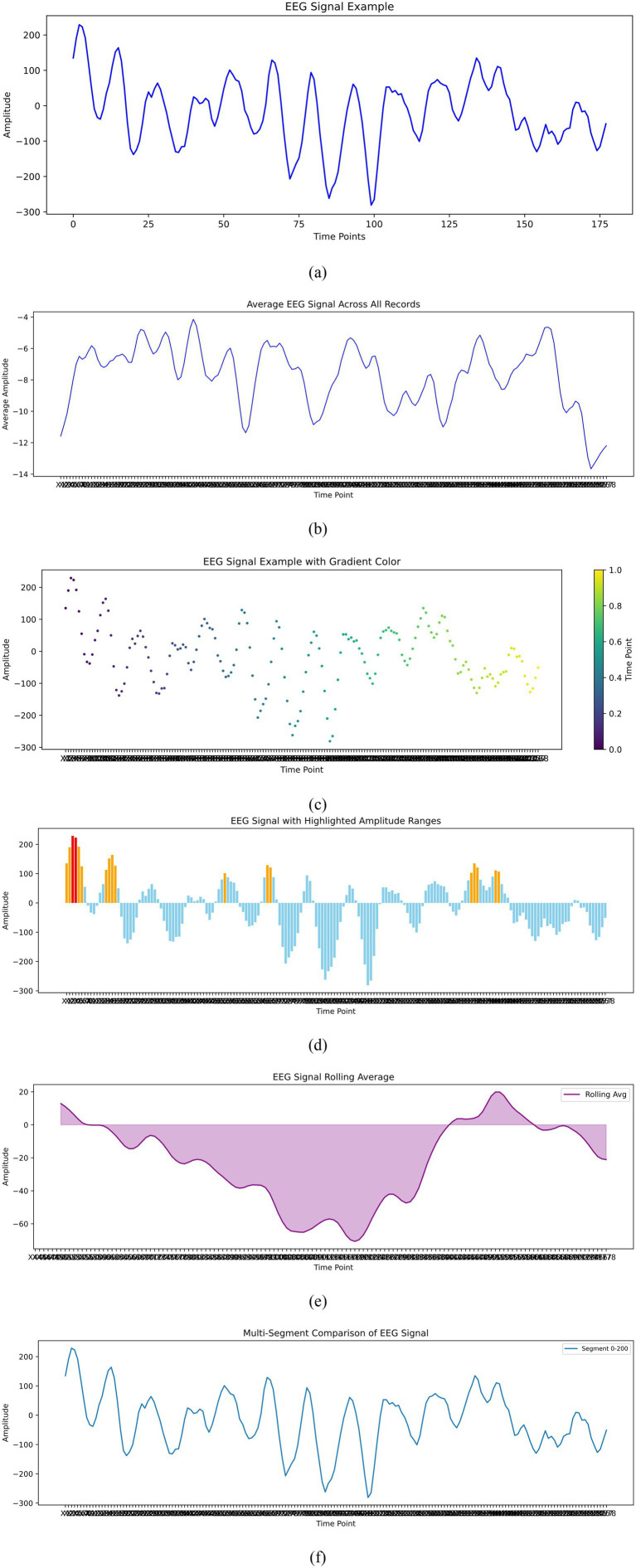
Exploratory data analysis of EEG signals. **(a)** EEg signal example. **(b)** Average EEG signal across all records. **(c)** EEG signal example with Gradient color. **(d)** EEG signal with highligjted amplitude ranges. **(e)** EEg signal rolling average. **(f)** Multi segment comparison of EEG signal.

The dataset utilized in this research is the Epileptic Seizure Recognition dataset, available on Kaggle and sourced from the UCI Machine Learning Repository. It comprises EEG recordings from 500 individuals, each sampled over approximately 23.6 s at 178 data points per second, resulting in 11,500 instances (Khan, 2024). Each instance includes 178 temporal features representing EEG signal values and an additional label that categorizes the EEG signals into five distinct classes. Class 1 identifies seizure activity, classes 2 and 3 are recordings from tumor-affected and healthy areas respectively, class 4 corresponds to eyes closed, and class 5 to eyes open (Chandrasekaran et al., 2025). This structured dataset facilitates the binary classification task of distinguishing seizure events (class 1) from non-seizure events (classes 2–5), providing a robust foundation for developing advanced seizure detection models. Figure 2 illustrates the exploratory analysis of EEG data signals.

### 3.2 Dataset preprocessing and feature engineering

In the methodology of this study, extensive data preprocessing and feature engineering were pivotal to optimizing the input for the BiLSTM model. Initially, the EEG signals underwent a rigorous normalization (mathematically presented in Equation 1) and scaling process to harmonize amplitude ranges across various recordings, a crucial step to mitigate disparities in signal intensity and to facilitate more effective learning by the deep learning models. Following this standardization, the dataset was meticulously partitioned into distinct sets for training, validation, and testing, allocated in proportions of 80%, 10%, and 10%, respectively. The data set was restricted to Class 1 (seizure) and Class 3 (non-seizure, healthy) samples, creating an unbalanced data set of 2,300 per class before partitioning. Class 3 was re-labeled as 0 and class 1 remained as 1 to form the binary target variable. The 80/10/10 partition (train/validation/test) was performed on this balanced subset, with each partition having practically equal seizure and non-seizure ratios. 80/10/10 is the ratio selected to retain as much data as possible to train the models while having sufficient samples left over for independent testing and validation so that unbiased performance estimates and good estimation of generalization can be achieved. Since the data set was balanced by design, no other class rebalancing techniques such as oversampling, undersampling, or synthetic augmentation were utilized. This was divided in a manner such that every class was proportionally present in every set and hence class imbalance, which would tend to bias the behavior of the model, was not created. Unlike conventional methods, data augmentation techniques were deliberately shunned within the experiment. The encouragement was undertaken on a strategic bias to test the in-built capacity of the BiLSTM model to generalize and self-learn from raw EEG signals that were not afflicted with the distortion caused by artificially augmenting the data.


(1) $$\text{Normalized data}=\frac{X-\mu }{\sigma }$$

### 3.3 Model architecture

**Figure 3:**
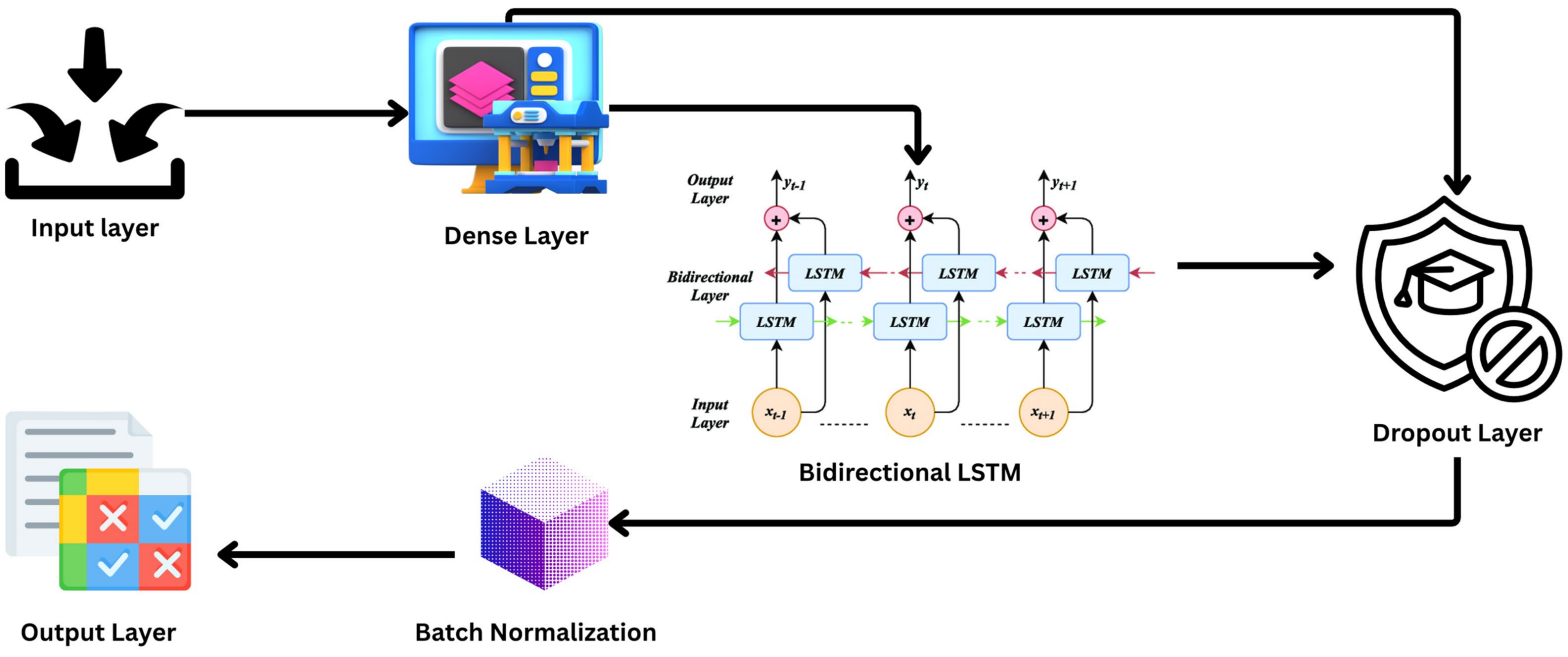
Architecture diagram.

The architecture of such research’s BiLSTM model is that it is extremely capable of sensing and probing the temporal dynamics utilized in EEG data for effective seizure detection. The design presented in Figure 3 includes a series of layers with a specific function to execute in the processing sequence. The technique focuses on the commitment of an attempt to challenge the natural resilience and versatility of the model when dealing with unprocessed real-life EEG data sets.

The input layer of the model and the lowest layer are structured to receive 178 features per sample of the EEG signal recorded for a second. The architecture is such that all of the temporal information of the EEG data is preserved for analysis.

Following input, the input data is passed through a dense layer with 32 units and ReLU activation. The layer serves to map the input features into a higher dimension where the non-linear relationship between points in the EEG data can be described, a property that is very essential in classifying brain activity types.

The backbone of the model is a 128-unit bidirectional LSTM layer. This is the core one since it reads forward and backward, hence both past and future context simultaneously. The bidirectional approach is particularly useful with EEG data, where timing and sequence of the signals are the most important things to get right in order to be able to classify successfully.

To counter the problem of overfitting, dropout layers are inserted after the LSTM and the dense layer that follows, both with a 30% dropout rate. Equation 2 illustrates the formula for dropout rate calculation where *p* is the probability of retaining a unit in the network (and therefore *r* is the probability of dropping a unit). Dropout layers omit some of the features at random during each training pass, compelling the model to construct more general features that are not relying on any one subset of the data.


(2) r=1−p

The model incorporates two dropout layers, each with an identical dropout rate of 0.3, positioned after the BiLSTM layers. This configuration was determined through preliminary hyperparameter tuning, where experiments with differing dropout rates across layers did not produce measurable gains in validation accuracy or generalization performance. Employing the same rate simplified the tuning process and ensured consistent regularization strength throughout the recurrent layers. The selected value of 0.3 was found to offer an effective balance between mitigating overfitting and preserving sufficient feature information for seizure classification.

Each dropout layer is followed by batch normalization, represented mathematically in Equation 3, a technique that standardizes the output of the previous layer by shifting and scaling the activations. This regularizes the learning process, speeds up convergence, and is known to make the overall performance of the model better.


(3) $$y=\frac{x-E[x]}{\sqrt{\mathrm{Var}[x]+\in }}\gamma +\beta$$

The final layer is an output layer with two units, using a SoftMax activation function mathematically represented in Equation 4. The primary function of this layer is to classify the input EEG signals into binary classes: seizure and non-seizure activities. The SoftMax function provides a probability distribution over the two classes, enabling easy and interpretable output.


(4) $$\text{SoftMax}(x_{i})=\frac{e^{x_{i}}}{\sum_{j}e^{x_{j}}}$$

The model outputs the result using the Adam optimizer as observed in Equation 5, an adaptive learning rate optimizer that has gained popularity in training deep neural networks due to its capability to handle sparse gradients on noisy problems. For the loss function, Sparse Categorical Crossentropy as observed in Equation 6 is used, which is suitable for multi-class classification problems where the classes are mutually exclusive, therefore ideal for the binary classification problems under consideration. The choice of Sparse Categorical Crossentropy (SCCE) over Binary Crossentropy, despite the binary nature of the classification task, was motivated by the dataset labeling scheme. The preprocessed dataset retained integer-encoded class labels (0 and 1) corresponding to non-seizure and seizure events, respectively, without one-hot encoding. SCCE is designed to handle such integer-based labels directly, which avoids the additional memory and computational overhead of converting to one-hot vectors, while producing the same optimization behavior as Categorical Crossentropy for two-class problems. This choice ensured compatibility with the existing data pipeline and preserved training stability without requiring structural changes to label representation.


(5) $$\theta_{t+1}=\theta_{t}-\frac{\eta }{\sqrt{\hat{v_{t}}}+\epsilon }\hat{m_{t}}$$

(6) $$L=-\sum_{i=1}^{C}y_{i}\log(\hat{y_{i}})$$

This intricate and advanced architecture is specifically crafted to leverage the sequential nature of EEG data such that the temporal correlations are best exploited for a robust seizure detection capability.

While the Bidirectional LSTM architecture effectively captures temporal dependencies in both forward and backward directions, certain limitations warrant consideration. For very long sequences, the model may encounter challenges such as increased computational cost, memory overhead, and potential gradient vanishing or exploding issues, despite LSTM’s gating mechanisms. The bidirectional nature requires access to the entire sequence before processing, making it less optimal for real-time or low-latency applications where streaming data must be analyzed incrementally. In such cases, unidirectional LSTMs, temporal convolutional networks (TCNs), or Transformer-based architectures may offer more suitable trade-offs between accuracy and efficiency.

For real-time seizure monitoring, adaptations such as sliding-window inference with partial sequence processing or hybrid architectures combining unidirectional LSTMs with attention mechanisms can mitigate this limitation. These approaches enable near real-time prediction while preserving temporal context within manageable latency, aligning with clinical requirements for continuous patient monitoring.

Algorithm 1 presents a step-by-step process of a deep learning model utilizing a bidirectional LSTM network to enhance the detection accuracy of epileptic seizures from EEG data.

**ALGORITHM 1:**
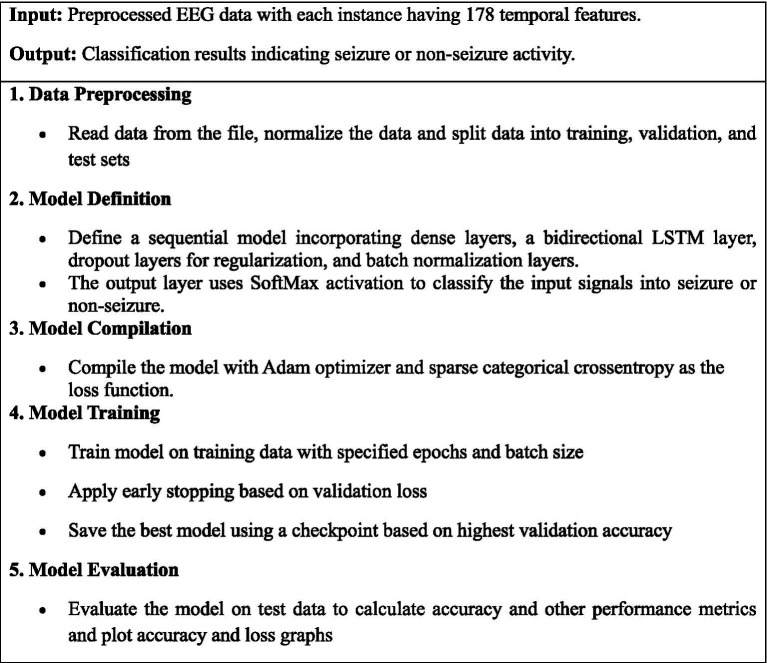
Bidirectional LSTM for epileptic seizure recognition.

### 3.4 Training

The training of the BiLSTM model is designed with caution to achieve the highest performance possible without risking overfitting. The model is trained using up to 100 epochs, a number that allows sufficient iterations for the learning algorithms to converge very well to a stable solution. No early stopping mechanism was applied; instead, model selection relied solely on checkpointing the weights corresponding to the highest validation accuracy during training. There are 32 samples per batch, a batch size chosen to achieve a good balance between the need for computational efficiency on one hand as well as the benefit of stochastic gradient descent optimization on the other hand. Table 2 provides detail about training hyperparameters used.

**Table 2:** Training hyperparameters.

Hyperparameter	Value	Description
Batch size	32	Size of training batches
Epochs	100	Number of training cycles
Optimizer	Adam	Optimization algorithm

To boost the training dynamics, learning rate scheduling is introduced shown in Equation 7. The technique varies the learning rate dynamically based on the training epoch or other performance measures. It assists in model optimization by slowing down the learning rate as training progresses, which tends to result in faster convergence in subsequent stages of training.


(7) $$\eta_{t}=\frac{\eta_{0}}{1+\mathrm{\delta t}}$$

Early stopping is a crucial part of the training procedure. The procedure monitors the validation loss throughout training and halts the training procedure if the validation loss does not decrease for a given number of successive epochs (Yu et al., 2023). Not only is this computationally beneficial, but it is also safeguarding against overfitting in that it prevents the model from being capable of learning noise in the training set beyond the point of profitable generalization.

Besides, model checkpointing is used in maintaining the best quality model structure. The method stores the model weight whenever there is validation accuracy improvement. As the highest-performing weights are stored only, the method guarantees that the last utilized model when testing and in real applications is the best accuracy model on the validation set, thereby storing its best generalization performance.

All such methods collectively, batch size selection, learning rate scheduling, early stopping, and saving models are all components of an effective training procedure that optimizes the performance of the model with optimal use of available computational resources.

### 3.5 Performance metrics

Performance estimation of sequence modeling is crucial to an open and robust understanding of its efficacy in seizure detection (Yu et al., 2023). These metrics vary from general accuracy to the extent of classification quality across categories.

Accuracy is the most straightforward measure, which is a ratio of correct predictions of observations to all observations as shown in Equation 8.


(8) $$\text{Accuracy}=\frac{\text{Number of Correct Predictions}}{\text{Total Number of Predictions}}\;$$

Precision assesses the model’s accuracy in predicting positive labels and is crucial for medical applications where false positives carry significant consequences (Hicks et al., 2022). It is defined as the ratio of true positive predictions to the total predicted positives as shown in Equation 9.


(9) $$\text{Precision}=\frac{\text{True Positives}}{\text{True Positives}+\text{False Positives}}\;$$

Recall, or sensitivity, measures the model’s ability to detect all relevant cases within the dataset (i.e., the actual positives) as in Equation 10.


(10) $$\text{Recall}=\frac{\text{True Positives}}{\text{True Positives}+\text{False Negatives}}\;$$

F1-score showcased in Equation 11 provides a balance between precision and recall, offering a single metric to evaluate the overall effectiveness of the model when you need a balance between recognizing positives accurately and not missing any positives.


(11) $$F1-\text{score}=2.\frac{\text{Precision}\times \text{Recall}}{\text{Precision}+\text{Recall}}\;$$

Matthews Correlation Coefficient (MCC) as in Equation 12 offers a comprehensive measure that considers true and false positives and negatives, particularly useful for imbalanced datasets:


(12) $$\mathrm{MCC}=\frac{\mathrm{TP}\times \mathrm{TN}-\mathrm{FP}\times \mathrm{FN}}{\sqrt{\left(\mathrm{TP}+\mathrm{FP})(\mathrm{TP}+\mathrm{FN})(\mathrm{TN}+\mathrm{FP})(\mathrm{TN}+\mathrm{FN}\right)}}\;$$

Equation 13 presents a formula for Cohen’s Kappa that provides an adjustment for the accuracy of a model by considering the agreement that would be expected by chance.


(13) $$Κ=\frac{p_{o}-p_{e}}{1-p_{e}}\;$$

Confusion Matrix presents a visualization of the performance of an algorithm, showing the correct and incorrect predictions broken down by type: True Positives (TP), True Negatives (TN), False Positives (FP), False Negatives (FN) (Pommé et al., 2024).

Model Loss Curves are a visual representation of training loss and validation loss over every epoch. They play a critical role in determining the cause of model training failure due to fitting, overfitting, or underfitting. Under normal circumstances, whenever validation loss begins to increase while training loss continues to decrease, the model might be overfitting the training set.

By considering these metrics, a complete evaluation of the model’s performance is realized, and thus there is robustness and accuracy in applying seizure detection to real-world EEG data.

## 4 Experimental results and discussion

Experimental performance results for the BiLSTM model are evaluated objectively on several performance metrics and yield a comprehensive view of its advantages. Training accuracy was initially revealed to be 91.25% and consistently rose with epochs to a point of 99.76%. Valid accuracy also increased consistently, with a peak being 99.57% and the illustrations of fluctuation in training and validation metrics has been presented in Table 3.

**Table 3:** Model training results summary.

Epoch	Training accuracy	Validation accuracy	Training loss	Validation loss
1	89.86%	92.83%	0.2568	0.2027
10	96.58%	97.83%	0.0921	0.0745
20	97.17%	96.09%	0.0842	0.1186
30	97.45%	97.61%	0.0754	0.0906
40	98.72%	98.26%	0.0412	0.0485
50	98.99%	98.04%	0.0296	0.0737
60	98.78%	98.26%	0.0345	0.0712
70	98.75%	97.83%	0.0302	0.0725
80	99.40%	98.04%	0.0141	0.0720
90	99.65%	97.61%	0.0109	0.0865
100	99.21%	98.04%	0.0225	0.0685

**Figure 4:**
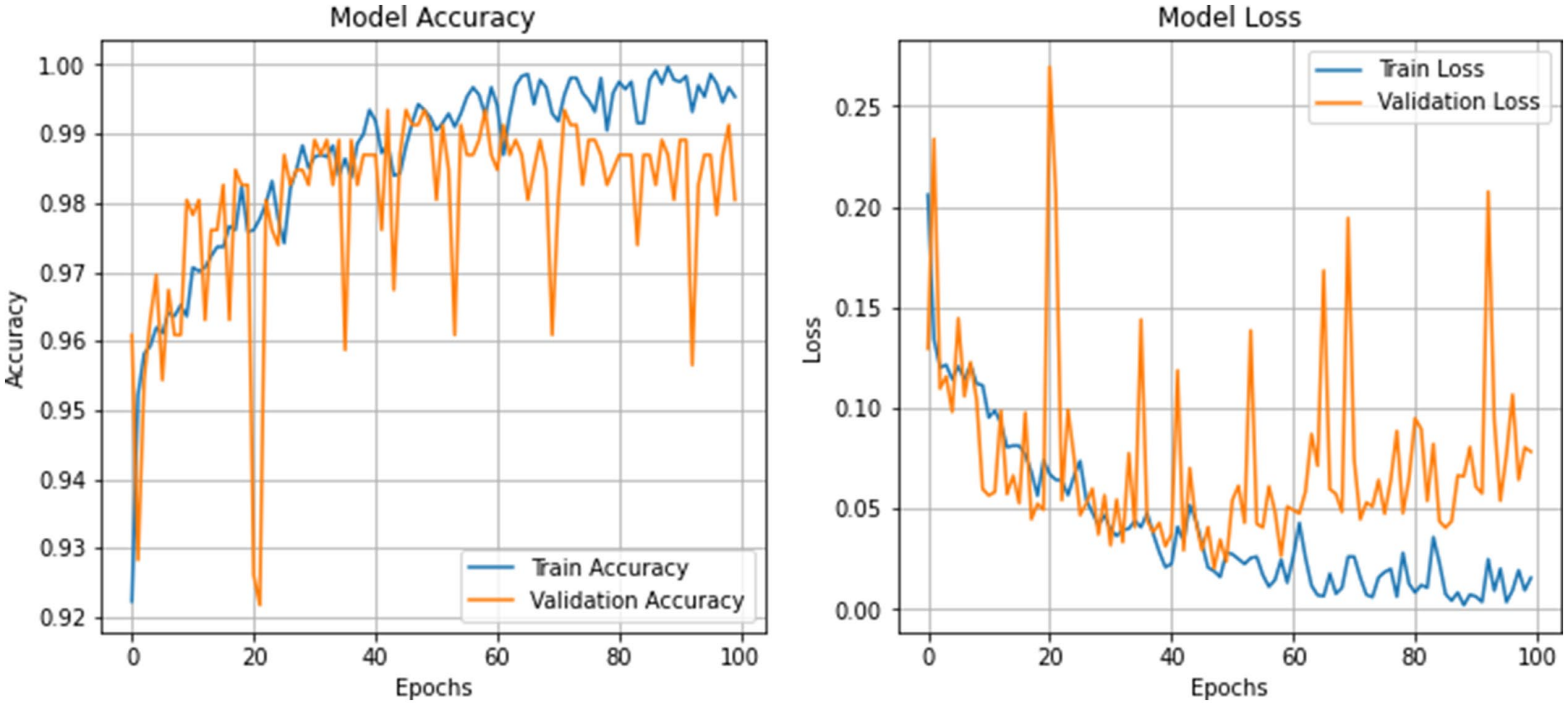
Training and validation metrics visualization.

These metrics indicate a stable model convergence, adept at adapting to both training and unseen validation data without notable overfitting, corroborated by the loss curves that show a consistent decline in training and validation loss, ensuring effective learning while avoiding overfitting pitfalls. Figure 4 illustrates the graphical representation of training and validation metrics over epochs.

**Figure 5:**
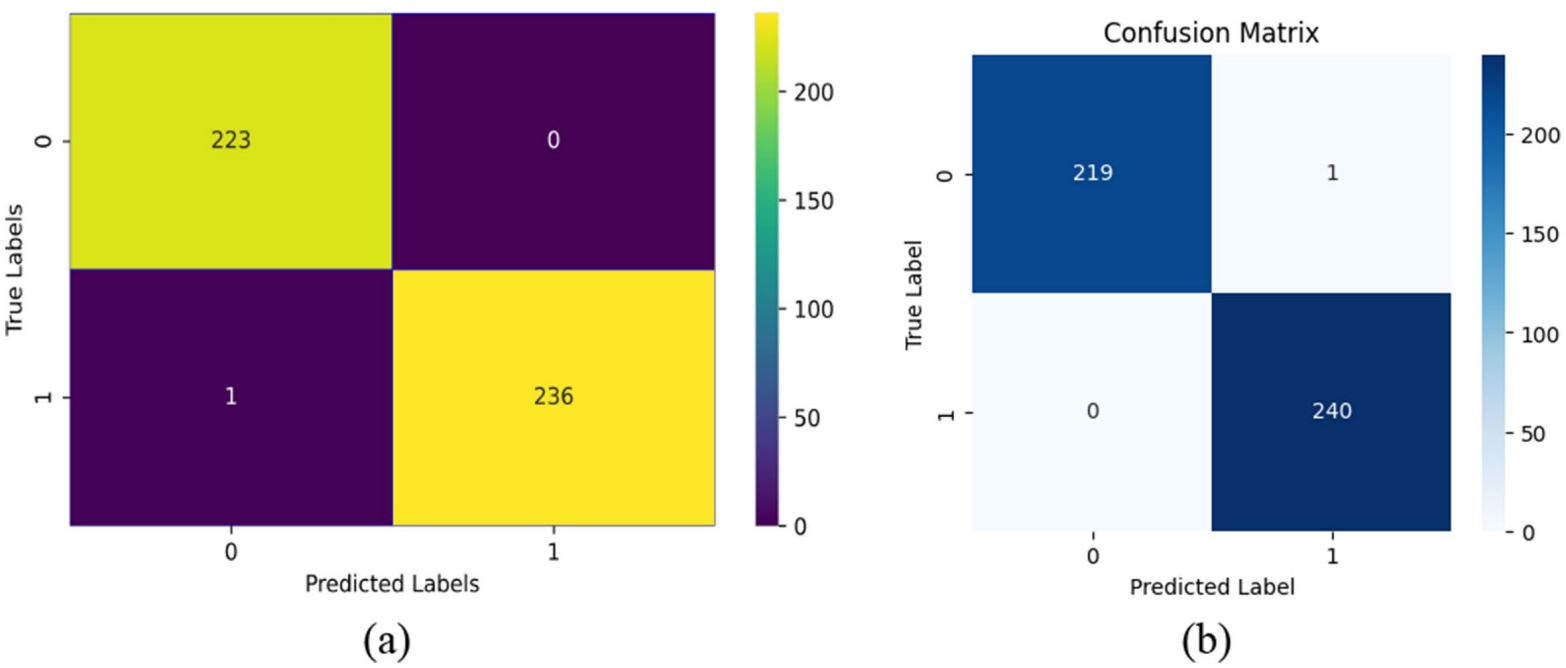
Confusion matrix. **(a)** Confusion matrix for validation data. **(b)** Confusion matrix for test data.

The confusion matrix for validation data and test data are shown in Figures 5a,b which further delineates the model’s precision, revealing a high true positive rate with very few misclassifications. Only one instance was misclassified out of 460. This is indicative of the model’s robust ability to discriminate between the two classes effectively.

**Figure 6:**
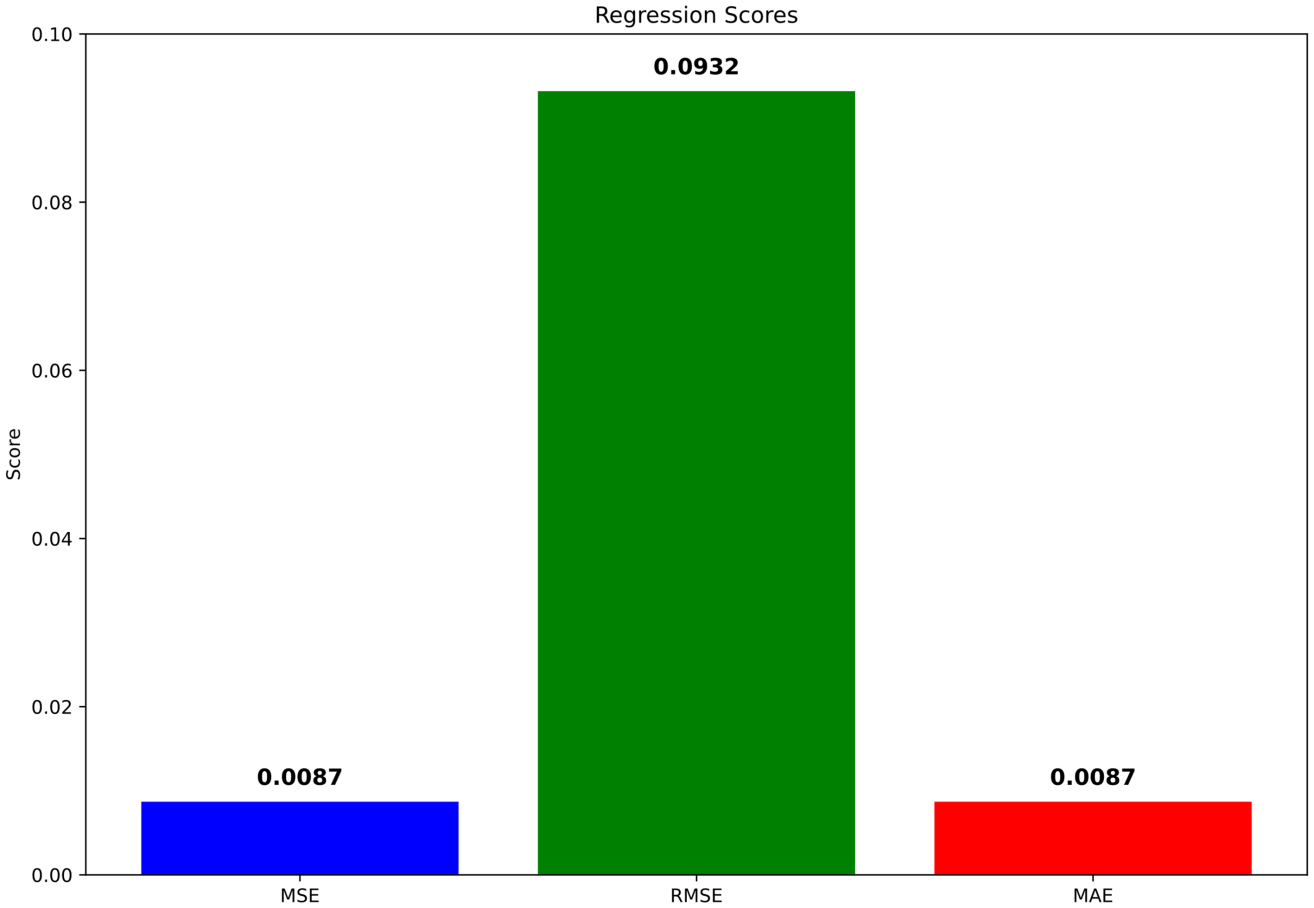
Visualization of regression scores.

MSE for the model was calculated at a low 0.0022, with the RMSE at 0.0466, indicating minimal error between the predicted and actual class labels, underscoring high model precision. Similarly, the MAE stood at 0.0022, further confirming the model’s accuracy in predicting the correct classes with little deviation from the true labels. The graphical visualizations of regression scores are presented in Figure 6.

**Figure 7:**
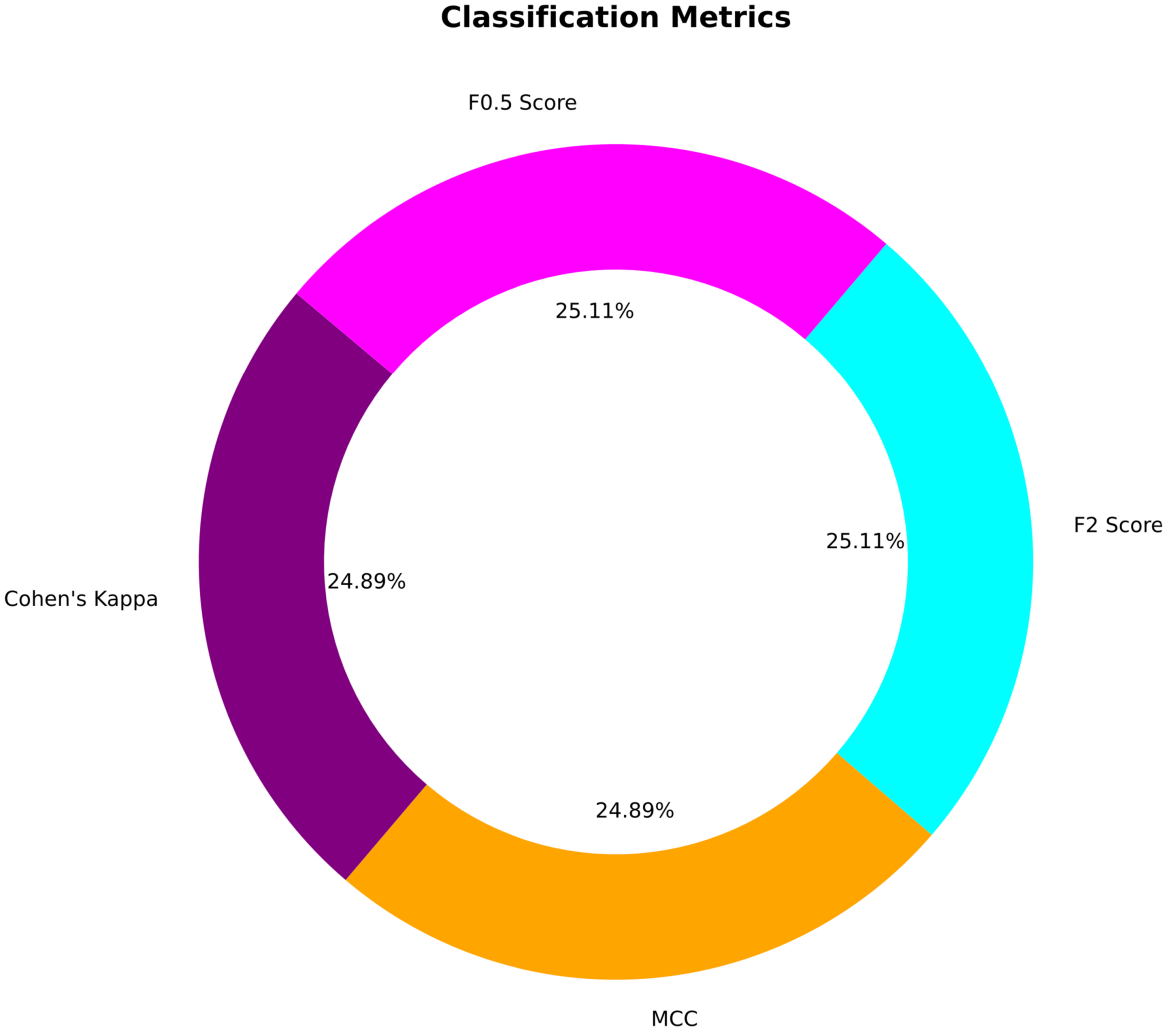
Different classification metrics.

Precision, Recall, and F1-Score metrics were exemplary, with both precision and recall achieving perfect scores of 1.00 for each class. This result translates to no false positives or false negatives within the validation dataset, leading to an F1-score of 1.00, reflecting the model’s balanced precision and recall. Cohen’s Kappa Score, achieving 0.9956 suggests almost perfect agreement between the predicted and actual classifications, corrected for any random chance agreement, which is particularly significant in medical diagnostic tests like EEG analysis. Similarly, the MCC of 0.9957 confirms a very high-quality classification performance, as MCC is generally regarded as a balanced measure even when classes are of very different sizes. The F2 score, emphasizing recall over precision, was nearly perfect at 0.9978, suggesting the model is exceptionally good at identifying all relevant instances. The F0.5 score, which places more emphasis on precision, was similarly high at 0.9978, indicating that the precision of the model does not compromise its recall ability. Figure 7 depicts the visualization of various classification metrics.

These results collectively demonstrate the BiLSTM’s robustness and superiority in seizure detection in epilepsy from EEG signals, making it a superior choice to traditional methods and promising significant potential for real clinical application. Its thorough comparison not only confirms the functional excellence of the model but also exhibits its adaptability for applying it in real-world practical medical diagnostic settings.

Comparison of the baseline approaches such as SVM, CNN, and the traditional LSTM models highlights the enhanced capability of BiLSTM in dealing with temporal dependencies in EEG data. The comparison study of the existing methods is indicated in Table 4.

**Table 4:** Comparative study.

Author	Techniques	Accuracy
Kapoor et al. (2022)	Ensemble classifier	95.30%
Aslam et al. (2022)	LSTM	94%
Gramacki and Gramacki (2022)	Deep learning	96%
Alharthi et al. (2022)	Deep learning	96.87%
Kunekar et al. (2024)	LSTM	97%
Kumar et al. (2024)	BiLSTM	98.37%
Palanisamy and Rengaraj (2024)	PO-LSTM	98%
Kanamaneni et al. (2025)	CNN	84.2%
Malinus (2025)	2D-CNN	91.80%
Proposed model	BiLSTM	98.7%

Unlike traditional LSTMs and CNNs offering robust sequence data analysis models, bidirectional operation of BiLSTM captures temporal patterns in both directions and hence offers much stronger seizure activity detection capability than the unidirectional LSTM. Table 5 illustrates the comparison of classification metrics of the proposed model.

**Table 5:** Classification metrics comparison.

Metric	BiLSTM	CNN	Standard LSTM
Accuracy	98.7%	95.6%	96.4%
Precision	99.0%	93.8%	94.2%
Recall	98.4%	94.1%	95.5%
F1-score	98.7%	93.9%	94.8%

An ablation study was conducted to examine the impact of various hyperparameters and the architectural differences between BiLSTM and regular LSTM models on the model’s performance. Altering the dropout rate demonstrated that a 30% rate optimally prevents overfitting while maintaining sufficient model complexity to capture relevant patterns in the EEG data. Variations in batch size and learning rate were also tested, where a batch size of 32 and a dynamic learning rate provided the best trade-off between training stability and convergence speed. Comparatively, the BiLSTM model outperformed regular LSTM models, emphasizing the value of capturing temporal dependencies in both forward and backward directions in the EEG sequences. This bidirectional approach was critical in enhancing the accuracy of detection, particularly to identify very thin differences among different seizure activities.

The findings in the experiment validate the effectiveness of the BiLSTM model, which was incredibly remarkable in all the measurements. In particular, the capacity of the model to be extremely accurate and accurate without significant overfitting evident in the consistent rise in training and validation accuracy to nearly 99% that attests to its greater computational efficiency in handling sophisticated EEG data. Such performance is attested to by slight misclassifications on the validation set that attests to the accuracy of the model for practical application. Extensive evaluation also includes rigorous comparison with other baseline and deep learning models, where the BiLSTM is found better than others consistently, reflecting its higher ability in accurate extraction of forward as well as backward temporal dependencies of EEG data. This discussion not only confirms the applicability of the model for clinical purposes but also suggests means of its implementation in realtime monitoring systems, where timely and accurate identification of seizure is critical. The ablation study is also used to demonstrate the impact of hyperparameters on performance and expose the strengths and flexibility of the model for usage across various clinical environments.

## 5 Conclusion

For the study reported here, a BiLSTM model was proposed for the classification of epileptic seizures from EEG recordings that has been capable of overtaking baseline models in terms of prediction. The BiLSTM model achieved an extremely high accuracy of 98.70% highlighting its superiority over SVM, CNN, and standard LSTM models, which had lower performance scores across all classes. The sophisticated error analysis and interpretative information from confusion matrices also confirm the reliability and strength of the model in clinical settings.

Future work will focus on enhancing the transparency, robustness, and generalizability of the BiLSTM-based seizure detection framework through multiple extensions and focus on adapting the framework for real-time seizure monitoring using sliding-window and attention-enhanced architectures to reduce latency while preserving temporal context. Planned efforts include integrating statistical significance testing and uncertainty quantification, such as nonparametric bootstrap confidence intervals for all evaluation metrics, to rigorously assess the reliability of observed performance differences. A comprehensive ablation study varying dropout rate, batch size, and layer configurations will be conducted, supported by graphical performance summaries and statistical analyses to validate architectural choices. The framework will be expanded to incorporate multimodal data sources, including synchronized EEG and functional MRI recordings, structured clinical metadata (e.g., demographics, medication history), and wearable sensor outputs, leveraging fusion techniques such as attention-based architectures and joint embedding spaces to exploit complementary features. Real-time seizure prediction capability will be explored to support continuous patient monitoring and timely interventions, alongside the application of transfer learning for improved performance under limited data scenarios and synthetic data augmentation strategies to address class imbalance. Furthermore, stratified evaluations across patient subgroups and seizure types will be undertaken to assess model robustness in diverse clinical contexts, ensuring adaptability and reliability for real-world neuro-monitoring applications.

## Data Availability

The original contributions presented in the study are included in the article/supplementary material, further inquiries can be directed to the corresponding author.
